# *N*-(2,4-Dichloro­phen­yl)-1,3-thia­zol-2-amine

**DOI:** 10.1107/S1600536812035301

**Published:** 2012-08-15

**Authors:** Ayesha Babar, Munawar Ali Munawar, M. Nawaz Tahir, Fateh Ullah, Muhammad Ilyas Tariq

**Affiliations:** a Institute of Chemistry, University of the Punjab, Lahore 54590, Pakistan; b University of Sargodha, Department of Physics, Sargodha, Pakistan; c Interdisciplinary Research Centre in Biomedical Materials, COMSATS Institute of Information Technology, Defence Road, Off Raiwind Road, Lahore, Pakistan; d University of Sargodha, Department of Chemistry, Sargodha, Pakistan

## Abstract

In the title mol­ecule, C_9_H_6_Cl_2_N_2_S, the mean planes of the benzene and thia­zole rings make a dihedral angle of 54.18 (8)°. In the crystal, mol­ecules are joined into dimers with an *R*_2_^2^(8) ring motif by pairs of N—H⋯N hydrogen bonds. These dimers are linked by C—H⋯Cl inter­actions into layers parallel to (011). The thia­zole rings form columns along the *c*-axis direction, with a centroid–centroid separation of 3.8581 (9) Å, indicating π–π inter­actions. An intra­molecular C—H⋯S contact also occurs.

## Related literature

For the synthesis and crystal structure of a related compound, see: Babar *et al.* (2012 ▶). For graph-set notation, see: Bernstein *et al.* (1995 ▶).
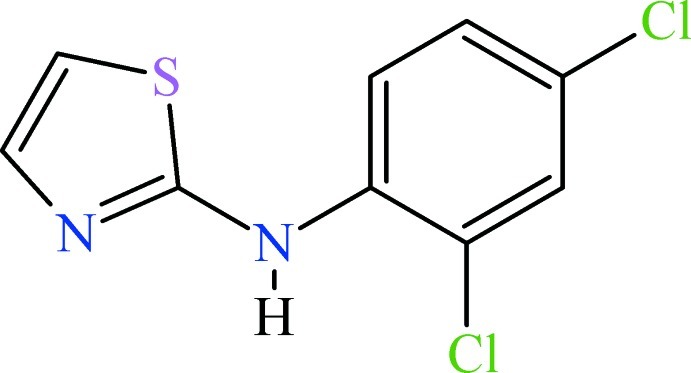


## Experimental

### 

#### Crystal data

C_9_H_6_Cl_2_N_2_S
*M* *_r_* = 245.12
Monoclinic, 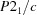

*a* = 13.0270 (9) Å
*b* = 10.1183 (6) Å
*c* = 7.7159 (5) Å
β = 91.974 (3)°
*V* = 1016.44 (11) Å^3^
*Z* = 4
Mo *K*α radiation
μ = 0.80 mm^−1^
*T* = 296 K
0.33 × 0.28 × 0.22 mm

#### Data collection

Bruker Kappa APEXII CCD area-detector diffractometer
Absorption correction: multi-scan (*SADABS*; Bruker, 2009 ▶) *T*_min_ = 0.778, *T*_max_ = 0.844
8039 measured reflections
2245 independent reflections
1957 reflections with *I* > 2σ(*I*)
*R*_int_ = 0.020

#### Refinement

*R*[*F* ^2^ > 2σ(*F* ^2^)] = 0.029
*wR*(*F* ^2^) = 0.080
*S* = 1.04
2245 reflections
127 parameters
H-atom parameters constrained
Δρ_max_ = 0.24 e Å^−3^
Δρ_min_ = −0.30 e Å^−3^

### 

Data collection: *APEX2* (Bruker, 2009 ▶); cell refinement: *SAINT* (Bruker, 2009 ▶); data reduction: *SAINT*; program(s) used to solve structure: *SHELXS97* (Sheldrick, 2008 ▶); program(s) used to refine structure: *SHELXL97* (Sheldrick, 2008 ▶); molecular graphics: *ORTEP-3 for Windows* (Farrugia, 1997 ▶) and *PLATON* (Spek, 2009 ▶); software used to prepare material for publication: *WinGX* (Farrugia, 1999 ▶) and *PLATON*.

## Supplementary Material

Crystal structure: contains datablock(s) global, I. DOI: 10.1107/S1600536812035301/yk2069sup1.cif

Structure factors: contains datablock(s) I. DOI: 10.1107/S1600536812035301/yk2069Isup2.hkl

Supplementary material file. DOI: 10.1107/S1600536812035301/yk2069Isup3.cml

Additional supplementary materials:  crystallographic information; 3D view; checkCIF report

## Figures and Tables

**Table 1:** Hydrogen-bond geometry (Å, °)

*D*—H⋯*A*	*D*—H	H⋯*A*	*D*⋯*A*	*D*—H⋯*A*
N1—H1⋯N2^i^	0.86	2.07	2.9302 (19)	174
C3—H3⋯Cl2^ii^	0.93	2.82	3.7483 (17)	173
C6—H6⋯S1	0.93	2.87	3.2056 (19)	103

Symmetry codes: (i) 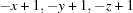; (ii) 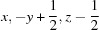.

**Fig. 1.**
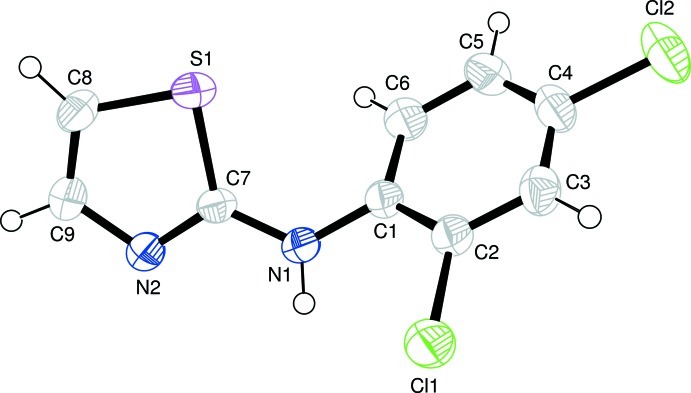
Molecular structure of the title compound showing the atom labelling scheme and displacement ellipsoids drawn at the 50% probability level.

**Fig. 2.**
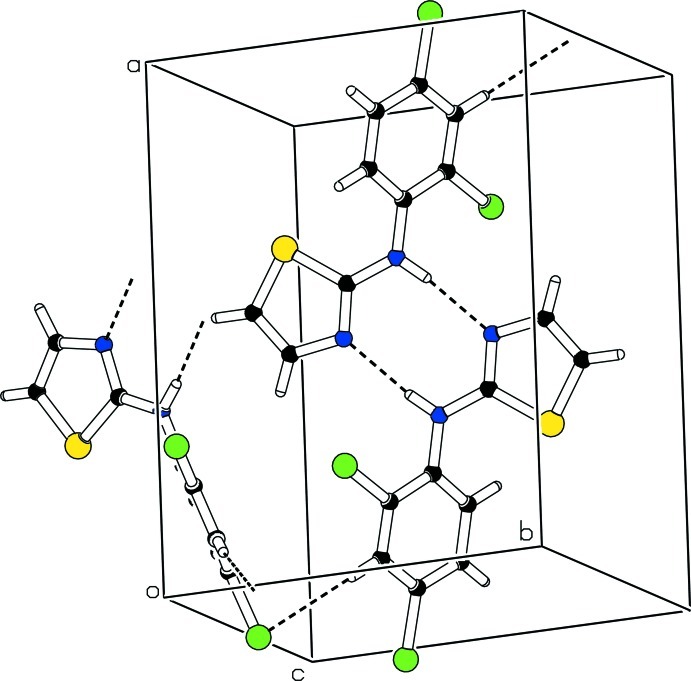
The partial packing showing molecular dimer linked to the anothers by C—H···Cl interactions.

## supplementary crystallographic information

### Comment

The title compound has been prepared in continuation to our ongoing project of synthesizing various derivatives of *N*-phenyl-1,3-thiazol-2-amine. We have recently published the synthesis and crystal structure of *N*-(2,4,6-trimethylphenyl)-1,3-thiazol-2-amine (Babar *et al.*, 2012) which is related to the title compound.

In the title compound (Fig. 1), the 1,3-dichlorobenzene group A (C1–C6/CL1/CL2) and 1,3-thiazol-2-amine group B (N1/C7/S1/C8/C9/N2) are planar with r.m.s. deviations of 0.009 Å and 0.030 Å, respectively. The dihedral angle between the planes of A and B is 53.28 (4)°. The molecules are joined into dimers by pairs of N—H···N hydrogen bonds (Table 1, Fig. 2), forming *R*_2_^2^(8) ring motif (Bernstein *et al.*, 1995). The dimers are further linked by C—H···Cl hydrogen bonds into layers parallel to (0 1 1). Thiazole rings form stacks along the *c* axis direction with intercentroid separation *Cg*···*Cg*^i^ [i = *x*, 3/2 - *y*, ±1/2 + *z*] of 3.8581 (9) Å, indicating π–π interactions.

### Experimental

A mixture of *N*-(2,4-dichlorophenyl)thiourea (1.00 g, 4.52 mmol) and 2-chloro-1,1-dimethoxyethane(0.93 g, 6.12 mmol) was dissolved in water- methanol mixture (1:2) (100 mL). A few drops of concentrated HCl were added and the reaction mixture was refluxed for 4 h. Water (100 ml) was added, and the mixture was neutralized with aqueous NaOH to pH=8. The resulting precipitate was filtered and washed with ice cold water. The crude product was recrystallized from chloroform - hexane mixture (1:2) to obtain white prisms.

### Refinement

The hydrogen atoms were positioned geometrically (C—H = 0.93 Å, N—H = 0.86 Å) and refined as riding with *U*_iso_(H) = 1.2*U*_eq_(C, N).

### Crystal data

**Table:**

C_9_H_6_Cl_2_N_2_S	*F*(000) = 496
*M**_r_* = 245.12	*D*_x_ = 1.602 Mg m^−^^3^
Monoclinic, *P*2_1_/*c*	Mo *K*α radiation, λ = 0.71073 Å
Hall symbol: -P 2ybc	Cell parameters from 912 reflections
*a* = 13.0270 (9) Å	θ = 1.6–27.2°
*b* = 10.1183 (6) Å	µ = 0.80 mm^−^^1^
*c* = 7.7159 (5) Å	*T* = 296 K
β = 91.974 (3)°	Prism, yellow
*V* = 1016.44 (11) Å^3^	0.33 × 0.28 × 0.22 mm
*Z* = 4	

### Data collection

**Table:**

Bruker Kappa APEXII CCD area-detector diffractometer	2245 independent reflections
Radiation source: fine-focus sealed tube	1957 reflections with *I* > 2σ(*I*)
Graphite monochromator	*R*_int_ = 0.020
Detector resolution: 7.80 pixels mm^-1^	θ_max_ = 27.2°, θ_min_ = 1.6°
ω scans	*h* = −16→16
Absorption correction: multi-scan (*SADABS*; Bruker, 2009)	*k* = −12→12
*T*_min_ = 0.778, *T*_max_ = 0.844	*l* = −9→9
8039 measured reflections	

### Refinement

**Table:**

Refinement on *F*^2^	Primary atom site location: structure-invariant direct methods
Least-squares matrix: full	Secondary atom site location: difference Fourier map
*R*[*F*^2^ > 2σ(*F*^2^)] = 0.029	Hydrogen site location: inferred from neighbouring sites
*wR*(*F*^2^) = 0.080	H-atom parameters constrained
*S* = 1.04	*w* = 1/[σ^2^(*F*_o_^2^) + (0.0406*P*)^2^ + 0.2925*P*] where *P* = (*F*_o_^2^ + 2*F*_c_^2^)/3
2245 reflections	(Δ/σ)_max_ < 0.001
127 parameters	Δρ_max_ = 0.24 e Å^−^^3^
0 restraints	Δρ_min_ = −0.30 e Å^−^^3^

### Special details

**Table:**

Geometry. Bond distances, angles *etc*. have been calculated using the rounded fractional coordinates. All su's are estimated from the variances of the (full) variance-covariance matrix. The cell e.s.d.'s are taken into account in the estimation of distances, angles and torsion angles
Refinement. Refinement of *F*^2^ against ALL reflections. The weighted *R*-factor *wR* and goodness of fit *S* are based on *F*^2^, conventional *R*-factors *R* are based on *F*, with *F* set to zero for negative *F*^2^. The threshold expression of *F*^2^ > σ(*F*^2^) is used only for calculating *R*-factors(gt) *etc*. and is not relevant to the choice of reflections for refinement. *R*-factors based on *F*^2^ are statistically about twice as large as those based on *F*, and *R*- factors based on ALL data will be even larger.

### Fractional atomic coordinates and isotropic or equivalent isotropic displacement parameters (Å^2^)

**Table:**

	*x*	*y*	*z*	*U*_iso_*/*U*_eq_	
Cl1	0.24571 (4)	0.37457 (5)	0.29544 (6)	0.0545 (2)	
Cl2	−0.05984 (3)	0.34681 (6)	0.74042 (8)	0.0662 (2)	
S1	0.33053 (3)	0.77775 (4)	0.66934 (5)	0.0405 (1)	
N1	0.35990 (10)	0.51946 (12)	0.57795 (18)	0.0403 (4)	
N2	0.49723 (10)	0.66580 (13)	0.57830 (18)	0.0400 (4)	
C1	0.26014 (11)	0.48156 (14)	0.6171 (2)	0.0353 (4)	
C2	0.19932 (12)	0.41107 (15)	0.4977 (2)	0.0369 (4)	
C3	0.10147 (12)	0.36979 (16)	0.5348 (2)	0.0434 (5)	
C4	0.06296 (12)	0.40070 (17)	0.6935 (2)	0.0451 (5)	
C5	0.12029 (14)	0.47052 (18)	0.8147 (2)	0.0505 (6)	
C6	0.21843 (14)	0.50928 (18)	0.7763 (2)	0.0463 (5)	
C7	0.40145 (11)	0.64084 (14)	0.60680 (18)	0.0326 (4)	
C8	0.43848 (14)	0.87355 (15)	0.6496 (2)	0.0455 (5)	
C9	0.51714 (14)	0.79861 (16)	0.6017 (2)	0.0462 (5)	
H1	0.39818	0.46092	0.53165	0.0483*	
H3	0.06225	0.32185	0.45384	0.0520*	
H5	0.09331	0.49142	0.92130	0.0606*	
H6	0.25772	0.55521	0.85926	0.0556*	
H8	0.44170	0.96415	0.66933	0.0545*	
H9	0.58188	0.83417	0.58503	0.0554*	

### Atomic displacement parameters (Å^2^)

**Table:**

	*U*^11^	*U*^22^	*U*^33^	*U*^12^	*U*^13^	*U*^23^
Cl1	0.0514 (3)	0.0636 (3)	0.0487 (3)	−0.0090 (2)	0.0058 (2)	−0.0158 (2)
Cl2	0.0351 (2)	0.0737 (3)	0.0905 (4)	−0.0031 (2)	0.0114 (2)	0.0243 (3)
S1	0.0421 (2)	0.0346 (2)	0.0453 (2)	0.0064 (2)	0.0081 (2)	−0.0047 (2)
N1	0.0325 (7)	0.0334 (6)	0.0554 (8)	−0.0019 (5)	0.0082 (6)	−0.0134 (6)
N2	0.0350 (7)	0.0333 (6)	0.0519 (8)	−0.0022 (5)	0.0059 (6)	−0.0070 (6)
C1	0.0319 (7)	0.0302 (7)	0.0438 (8)	0.0008 (6)	0.0034 (6)	−0.0011 (6)
C2	0.0365 (8)	0.0338 (7)	0.0405 (8)	0.0009 (6)	0.0019 (6)	−0.0005 (6)
C3	0.0351 (8)	0.0396 (8)	0.0550 (10)	−0.0030 (6)	−0.0047 (7)	0.0039 (7)
C4	0.0319 (8)	0.0420 (9)	0.0619 (11)	0.0019 (7)	0.0075 (7)	0.0141 (8)
C5	0.0470 (10)	0.0526 (10)	0.0530 (10)	0.0006 (8)	0.0170 (8)	−0.0013 (8)
C6	0.0454 (9)	0.0480 (9)	0.0458 (9)	−0.0049 (7)	0.0059 (7)	−0.0078 (7)
C7	0.0342 (8)	0.0313 (7)	0.0325 (7)	0.0025 (6)	0.0021 (6)	−0.0045 (5)
C8	0.0567 (10)	0.0289 (7)	0.0511 (9)	−0.0023 (7)	0.0071 (8)	−0.0040 (7)
C9	0.0449 (9)	0.0357 (8)	0.0583 (10)	−0.0084 (7)	0.0080 (8)	−0.0052 (7)

### Geometric parameters (Å, º)

**Table:**

Cl1—C2	1.7327 (16)	C2—C3	1.381 (2)
Cl2—C4	1.7399 (16)	C3—C4	1.375 (2)
S1—C7	1.7425 (15)	C4—C5	1.372 (2)
S1—C8	1.7191 (18)	C5—C6	1.379 (3)
N1—C1	1.3979 (19)	C8—C9	1.337 (2)
N1—C7	1.3574 (19)	C3—H3	0.9300
N2—C7	1.2992 (19)	C5—H5	0.9300
N2—C9	1.379 (2)	C6—H6	0.9300
N1—H1	0.8600	C8—H8	0.9300
C1—C6	1.389 (2)	C9—H9	0.9300
C1—C2	1.391 (2)		
			
C7—S1—C8	88.89 (7)	C1—C6—C5	121.76 (15)
C1—N1—C7	125.53 (13)	S1—C7—N2	114.44 (11)
C7—N2—C9	110.16 (13)	S1—C7—N1	123.54 (11)
C7—N1—H1	117.00	N1—C7—N2	121.87 (13)
C1—N1—H1	117.00	S1—C8—C9	109.99 (12)
N1—C1—C6	122.05 (14)	N2—C9—C8	116.50 (16)
N1—C1—C2	120.67 (14)	C2—C3—H3	121.00
C2—C1—C6	117.26 (14)	C4—C3—H3	121.00
C1—C2—C3	121.78 (14)	C4—C5—H5	120.00
Cl1—C2—C3	118.39 (12)	C6—C5—H5	120.00
Cl1—C2—C1	119.83 (12)	C1—C6—H6	119.00
C2—C3—C4	118.92 (15)	C5—C6—H6	119.00
C3—C4—C5	121.14 (15)	S1—C8—H8	125.00
Cl2—C4—C3	118.70 (12)	C9—C8—H8	125.00
Cl2—C4—C5	120.15 (13)	N2—C9—H9	122.00
C4—C5—C6	119.13 (15)	C8—C9—H9	122.00
			
C8—S1—C7—N1	−174.25 (13)	C6—C1—C2—Cl1	179.25 (12)
C8—S1—C7—N2	1.39 (12)	C6—C1—C2—C3	−0.1 (2)
C7—S1—C8—C9	−0.80 (12)	N1—C1—C6—C5	−179.38 (15)
C7—N1—C1—C2	134.53 (16)	C2—C1—C6—C5	−0.9 (2)
C7—N1—C1—C6	−47.0 (2)	Cl1—C2—C3—C4	−178.59 (13)
C1—N1—C7—S1	−9.7 (2)	C1—C2—C3—C4	0.8 (2)
C1—N1—C7—N2	174.96 (14)	C2—C3—C4—Cl2	−179.29 (12)
C9—N2—C7—S1	−1.53 (17)	C2—C3—C4—C5	−0.5 (3)
C9—N2—C7—N1	174.19 (14)	Cl2—C4—C5—C6	178.34 (14)
C7—N2—C9—C8	0.9 (2)	C3—C4—C5—C6	−0.5 (3)
N1—C1—C2—Cl1	−2.2 (2)	C4—C5—C6—C1	1.2 (3)
N1—C1—C2—C3	178.44 (14)	S1—C8—C9—N2	0.13 (18)

### Hydrogen-bond geometry (Å, º)

**Table:**

*D*—H···*A*	*D*—H	H···*A*	*D*···*A*	*D*—H···*A*
N1—H1···N2^i^	0.86	2.07	2.9302 (19)	174
C3—H3···Cl2^ii^	0.93	2.82	3.7483 (17)	173
C6—H6···S1	0.93	2.87	3.2056 (19)	103

Symmetry codes: (i) −*x*+1, −*y*+1, −*z*+1; (ii) *x*, −*y*+1/2, *z*−1/2.
